# Comparison of Variable Selection Methods for Time-to-Event Data in High-Dimensional Settings

**DOI:** 10.1155/2020/6795392

**Published:** 2020-07-01

**Authors:** Julia Gilhodes, Florence Dalenc, Jocelyn Gal, Christophe Zemmour, Eve Leconte, Jean-Marie Boher, Thomas Filleron

**Affiliations:** ^1^Department of Biostatistics, Institut Claudius Regaud, IUCT-O, Toulouse, France; ^2^Department of Medical Oncology, Institut Claudius Regaud, IUCT-O, Toulouse, France; ^3^Department of Biostatistics, Centre Antoine-Lacassagne, Nice, France; ^4^Department of Clinical Research and Investigation, Biostatistics and Methodology Unit, Institut Paoli-Calmettes, Aix-Marseille University, INSERM, IRD, SESSTIM, Marseille, France; ^5^TSE-R, Université de Toulouse, France

## Abstract

Over the last decades, molecular signatures have become increasingly important in oncology and are opening up a new area of personalized medicine. Nevertheless, biological relevance and statistical tools necessary for the development of these signatures have been called into question in the literature. Here, we investigate six typical selection methods for high-dimensional settings and survival endpoints, including LASSO and some of its extensions, component-wise boosting, and random survival forests (RSF). A resampling algorithm based on data splitting was used on nine high-dimensional simulated datasets to assess selection stability on training sets and the intersection between selection methods. Prognostic performances were evaluated on respective validation sets. Finally, one application on a real breast cancer dataset has been proposed. The false discovery rate (FDR) was high for each selection method, and the intersection between lists of predictors was very poor. RSF selects many more variables than the other methods and thus becomes less efficient on validation sets. Due to the complex correlation structure in genomic data, stability in the selection procedure is generally poor for selected predictors, but can be improved with a higher training sample size. In a very high-dimensional setting, we recommend the LASSO-pcvl method since it outperforms other methods by reducing the number of selected genes and minimizing FDR in most scenarios. Nevertheless, this method still gives a high rate of false positives. Further work is thus necessary to propose new methods to overcome this issue where numerous predictors are present. Pluridisciplinary discussion between clinicians and statisticians is necessary to ensure both statistical and biological relevance of the predictors included in molecular signatures.

## 1. Introduction

With the advent of genomic technologies, personalized medicine is becoming a major concern in oncology [1]. An important issue is improving the management of cancer patients by identifying profiles of patients at risk of relapse. To assist clinicians in prognosis assessment and therapeutic decision-making, multigene signatures have been developed to stratify cancer patients into different risk groups. Since the publication of the first gene signature, many prognostic multigene signatures have been extensively studied, but only some of them have been successfully implemented in clinical practice [2–4]. Among the latter, the most promising are gene signatures commercially available for early breast cancer that predict the risk of metastatic relapse [5–7]. Nevertheless, reproducibility is lacking for many published gene signatures currently not implemented in clinical practice. Much debate is ongoing in both medical and statistical literature to explain this high rate of failure for prognostic signatures. Biological relevance appears questionable since random signatures or signatures unrelated to cancer (e.g., signatures pertaining to the effect of postprandial laughter or mouse social defeat) have been shown to be significantly associated to overall survival. It is even more surprising that many random signatures can outperform most breast cancer signatures [8]. Several authors have suggested that the selected sets of genes are not unique and are strongly influenced by the subset of patients included in the training cohort [9, 10] and by the variable selection procedures [11–14]. For low-dimensional data, the reference method to study associations with time-to-event endpoints is the Cox proportional hazards model. In the context of high-dimensional data (number of covariates > >number of observations), the Cox model may be nonidentifiable. Extensions, based on boosting or penalized regression, are proposed in the literature to overcome these hurdles [15–18], as they shrink the regression coefficients towards zero. Alternatively to the Cox extensions, methods based on random forests have been adapted for survival analysis [19]. This nonparametric method—random survival forest (RSF)—combines multiple decision trees built on randomly selected subsets of variables. Since feature selection methods are questioned, it seems important to thoroughly assess and compare existing strategies that are significant components in prognostic signature development. Many studies were interested in false discovery rates or prognostic performances achieved by multiple variable selection methods and compared them on simulated or real datasets [20–23]. However, the impact of the training set on the stability of the results was only assessed by Michiels et al. [9] on a binary endpoint with a selection based on Pearson's correlation and did not evaluate most recent approaches.

The main objective of this publication is to compare six typical different feature selection methods which are commonly used for high-dimensional data in the context of survival analysis. For this purpose and as recommended in the literature [24], a simulation study is performed, with special focus on variable selection and prediction performance according to multiple data configurations (sample size of the training set, number of genes associated with survival). Feature selection methods are then applied on published data to explore stability and prognostic performances in a real breast cancer dataset.

## 2. Material and Methods

### 2.1. Feature Selection Methods

#### 2.1.1. Limits of the Cox Model in High-Dimensional Data

In low-dimensional data, the semiparametric Cox proportional hazards model is commonly used to study the relationship between covariates and time-to-event endpoints. The *β* regression coefficients related to the Z genes are estimated by maximizing the partial log-likelihood *l*(*β*) without it being necessary to model the baseline hazard:

(1) $$\begin{array}{c} l\left(\beta \right)=\overset{n}{\underset{i=1}{\sum }}\delta_{i}\left\{\beta^{T}Z_{i}-\log\left[\underset{j\in R\left(T_{i}\right)}{\sum }\exp\left(\beta^{T}Z_{j}\right)\right]\right\}, \end{array}$$

where *n* is the number of observations, *δ*_*i*_ is the event indicator for patient *i*, *β* is the regression parameter vector, *Z* is the vector of covariates, and *R*(*T*_*i*_) denotes the set of patients at risk before time *T*_*i*_. However, in the case of high-dimensional data, this model fails to be identifiable. Several methods have been proposed to handle such a case with a number of predictors *p* > >number of patients*n*. In this study, three kinds of computational methods were used to train the models: penalized Cox regression models, component-wise boosting for the Cox model, and RSF.

#### 2.1.2. Penalized Approaches

Penalized Cox regression models make it possible to simultaneously perform coefficient estimation and variable selection. The Least Absolute Shrinkage and Selection Operator (LASSO) is an L1-norm regularization method [17]. Coefficients are estimated by maximizing a penalized log partial-likelihood:

(2) $$\begin{array}{c} l\left(\beta \right)-\lambda \overset{p}{\underset{j=1}{\sum }}\left|\beta_{j}\right|, \end{array}$$

with a tuning parameter *λ*. The choice of this shrinkage parameter is challenging and is generally obtained by maximizing the cross-validated log-likelihood function (LASSO-cvl). Certain recent publications have highlighted the fact that the cvl method for choosing *λ* results in selecting a high number of false positives. Ternès et al. [16] proposed an extension of the cross-validation approach, denoting penalized cross-validated log-likelihood (pcvl), and compared its performances to other existing extensions (adaptive LASSO, percentile LASSO, etc.). This approach, trading off between goodness-of-fit and parsimony of the mode, leads to the selection of fewer genes by applying a more stringent selection criterion. LASSO-pcvl results in the best compromise between a decline in the false discovery rate and no large increase in the false-negative rate and thus was included in our comparison study. On the other hand, LASSO suffers, however, from some limitations. The number of features selected is bounded to the number of patients, and in the case of highly correlated predictors, LASSO tends to select only one of these features, resulting in a random selection in this group of features. This may not be desirable given that genes operating in the same biological pathway may be highly correlated, so taking this combination into account may be relevant.

To alleviate these limitations, Zou and Hastie [18] proposed the Elastic Net method—a penalized regression with the combination of the L1-norm and the L2-norm penalties. The additional L2-regularization term makes it possible to promote a grouping effect, thus removing the limitation of the number of selected variables. Coefficients are estimated by maximizing the partial log-likelihood *l*(*β*) subject to the penalty:

(3) $$\begin{array}{c} \lambda \left(\left(1-\alpha \right)\overset{p}{\underset{j=1}{\sum }}\left|\beta_{j}\right|+\alpha \overset{p}{\underset{j=1}{\sum }}\beta_{j}^{2}\right). \end{array}$$

The regularization parameter *λ* and mixing parameter *α* are estimated by cross-validation. *For* a more flexible alternative to LASSO without having to estimate two parameters, it was proposed to set the default value at 0.5 for the mixing parameter *α* and to estimate only the tuning parameter *λ* by cross-validation [11]. A stability selection approach based on subsampling in combination with the Elastic Net algorithm may be performed (BSS Enet) [25, 26], applying the algorithm to subsamples obtained by bootstrapping. The proportion of subsamples in which the biomarker is selected in the model corresponds to the selection probability for this particular biomarker. Only genes selected with an occurrence frequency equal to or larger than *σ* (with *σ* ∈ [0.5; 1]) are included in the final model.

We used the routine implemented in *the glmnet* package to determine the optimal penalty for LASSO-cvl and Elastic Net [27], and the *biospear* package for LASSO-pcvl. The penalty parameter *λ* was chosen based on the 10-fold cross-validation, and the mixing parameter *α* was set at 0.5 for Elastic Net regression and BSS Enet. The threshold *σ* selection probability was arbitrarily set at 0.5 for BSS Enet.

The Ridge regression was not included in this study since, unlike L1 regression or a mixture of L1 and L2, L2 penalty (*α* = 1) tends to minimize the parameters without providing variable selection.

#### 2.1.3. Boosting Algorithm

Like forward stepwise regression, boosting is an iterative method which starts from a null model and then adapts only one coefficient at a time, the one that maximizes a penalized partial log-likelihood, including a boosting penalty [15]. Previous boosting steps were incorporated in the penalized partial log-likelihood as an offset for the next step. This variable selection method was implemented in the R package *CoxBoost*. A 10-fold cross-validation was performed to find the optimal number of boosting steps via cv. *CoxBoost*, with a boosting penalty chosen via the *optimCoxBoostPenalty* function.

#### 2.1.4. Random Survival Forests

Random forests are nonparametric variable selection methods that have been extended for survival data [19]. The general algorithm consists in drawing bootstrap samples from the dataset, growing a tree for each of them and finally averaging predictions. For each node, a subset of predictor variables was selected as candidates to split data into two daughter nodes, according to the log-rank splitting rule. Then, the best split feature from this subset is used to split the node. Variable hunting was then used for variable selection [28]. Survival trees were built according to the parameters recommended by the authors in the case of high-dimensional data, using the R *randomForestSRC* package.

### 2.2. Simulated Datasets

Datasets were simulated with different sample sizes (*N* = 500, 750, or 1000) and a predetermined number *p* of 1500 normally distributed covariates. For each sample size, we generated survival times following an exponential regression model (with baseline equal to 1). Three scenarios with different numbers of truly prognostic biomarkers (*q* = 0, 12, or 50) were investigated. Regression parameters were fixed at -0.11 or -0.22 (resulting in more or less important effects of the true predictors), with hazard ratios of 0.9 or 0.8, respectively. To simulate a biologically relevant gene correlation structure, predictors were divided into subgroups of correlated covariates, with an autoregressive correlation structure [29]: (*σ*_*ij*_^2^ = *ρ*^|*i* − *j*|^). The parameter *ρ* was set to 0.6 after a review of the literature [16, 30, 31] and analysis of several published datasets like METABRIC and TCGA cohorts. Censoring times were, respectively, simulated using the uniform distribution (U [3, 5]), leading to censoring rates from 10 to 30%. Simulation parameters are summarized in Table 1.

### 2.3. Application on a Published Dataset

A public breast cancer dataset with available survival and gene expression data for 614 patients was used as an application. This dataset was extracted from GitHub (http://github.com/Oncostat/biospear/) to obtain the same data version as Ternès et al. [16]. The endpoint of interest was the distant recurrence-free survival, with a censoring rate of 78.2%. Probes were previously prefiltered according to an interquartile range greater than 1 in order to reduce the number of predictors with low variance across the samples (*p* = 1689).

### 2.4. Comparison of Variable Selection Methods

A resampling strategy was performed to evaluate the six selection methods: LASSO-cvl, LASSO-pcvl, Elastic Net, BSS Enet, CoxBoost, and RSF. Following the strategy used by Michiels et al., both simulated and real datasets were randomly split into 100 training and validation sets with different sample sizes for the training set (1/2 and 2/3 of the overall dataset) [9, 12]. For each training set, the different selection methods were applied to select significant genes and create a risk score for prediction. For the penalized and boosting approaches, the risk score was based on the linear predictor given by the Cox model. For RSF, the risk score was based on the average over the trees of the cumulative hazard estimations computed from the bootstrap samples which exclude this patient in order to reduce optimism bias. Models previously developed were then applied on the respective validation sets.

Based on the feature selection during the training stage, variable selection methods were compared in terms of number of selected genes and stability of the signatures, measured by the frequency of selection of each gene among the 100 training sets. For simulated datasets, both the FDR and the false-negative rate (FNR) could be computed. They corresponded, respectively, to the rates of inactive genes selected and the rates of true biomarkers missed by variable selection methods. Prognostic performances were then evaluated on the validation set by the Brier and C-index scores for risk scores, both implemented in the R *pec* package [32]. The integrated Brier score (IBS) is the area under the prediction error curve (i.e., the quadratic difference between the observed response and the predicted probability over time). IBS is tending to 0 for a perfect model. The C-index measures the discriminant ability of the model; its interpretation is similar to the classical area under the Receiver Operating Characteristic (ROC) curve. All analyses were implemented with R-3.2.4.

## 3. Results

### 3.1. Simulated Datasets

#### 3.1.1. Variable Selection

The number of genes selected by selection methods for each sample size and scenario is presented in Table 2. For the null scenario (*q* = 0, i.e., no active biomarker), BSS Enet and RSF selected genes in all simulated cases, whereas the other methods tended to select no predictors. LASSO-cvl and Elastic Net performed the best with the smallest FDR (respectively, range = [0.23‐0.55, 0.24‐0.56]). Obviously, an FDR of 1 for BSS Enet and RSF was observed (Table 3). For alternative scenarios (*q* = 12, 50), the number of selected genes by boosting and penalized approaches increased when the sample size of the training set increased. On the contrary, for RSF, the number of genes was inversely correlated to the training set sample size. The FDR and FNR were, respectively, minimized by the LASSO-pcvl and Elastic Net approaches. The FDR of BSS Enet was reduced compared to that of Elastic Net (25% decrease in median over different scenarios), but sometimes involved a small FNR increase. Stability results (Table 4) show that the latter increased with the sample size of the training set and decreased when the number of true predictors increased. When the sample size of the training set was maximal (*N*_training_ > 500), the occurrence frequency was approximately 100% for more than 50% of the selected predictors, except for RSF with the worst selection stability (median occurrence frequency (%) = 42; range = [18‐74]). If the training sample size is not sufficient to select a large number of predictors, stability tends to decrease in the presence of more true predictors. Some true predictors had poor occurrence frequency, and false predictors may have been more stable in our *in silico* study. A poor intersection was observed between predictors selected by each approach (Table 5). Among concordant selected predictors, there were few true positives. This proportion can be far less than 50%, particularly when the number of true predictors is moderate (*q* = 12) and where large training sample sizes are used.

#### 3.1.2. Prognostic Performances

**Figure 1:**
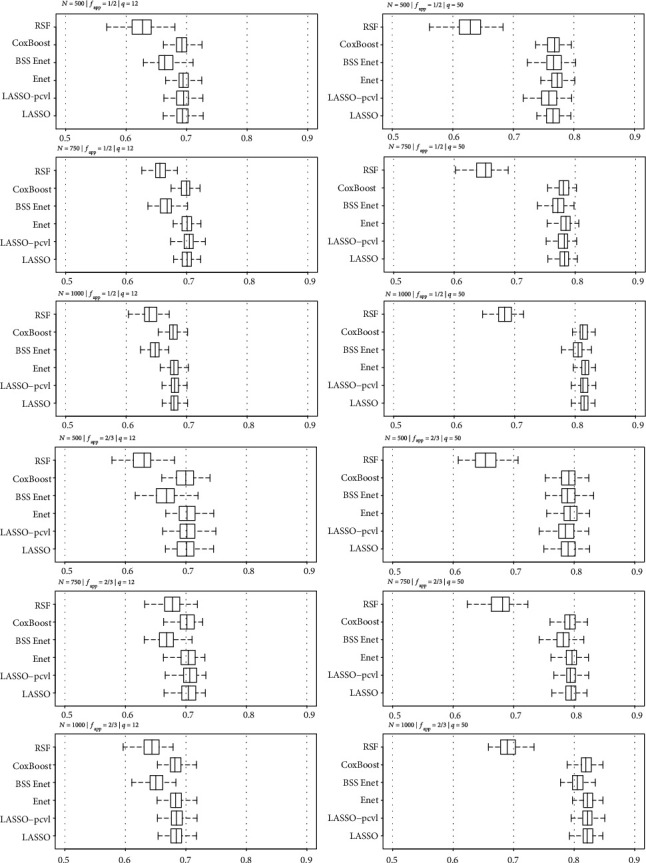
C-index associated to risk score for each selection method and fraction of the training data (*f*_app_) according to the sample sizes (a) *N* = 500, (b) *N* = 750, and (c) *N* = 1000 for simulated datasets with *q* = 12.

**Figure 2:**
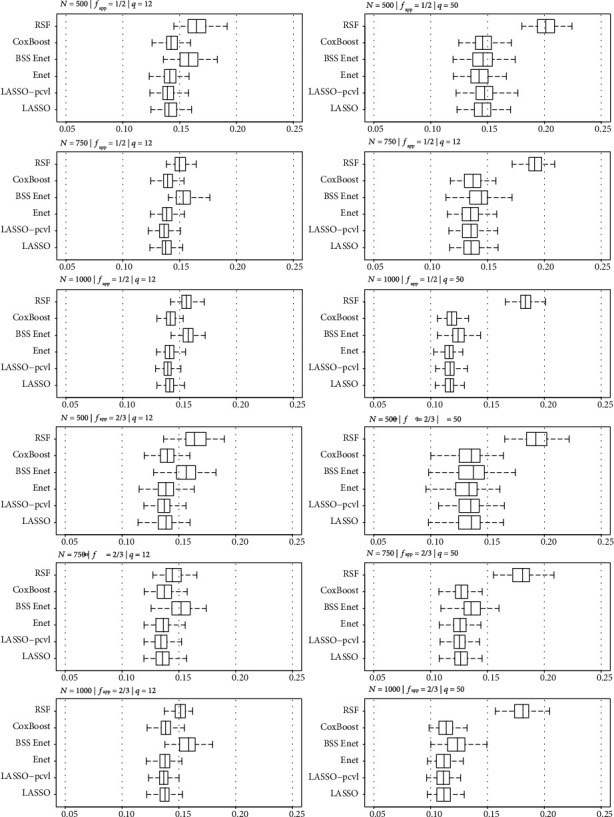
Brier score associated to risk score for each selection method and fraction of the training data (*f*_app_) according to the sample sizes (a) *N* = 500, (b) *N* = 750, and (c) *N* = 1000 for simulated datasets with *q* = 12.

For a low number of predictors (*q* = 12), the penalized and boosting approaches obtained better prognostic performances with higher C-index and lower IBS than RSF. For this scenario, prognostic performances tended to decrease when sample size increased. In contrast for *q* = 50, all approaches except RSF presented similar performances, and prognostic performances increased with sample size (Figures 1 and 2).

### 3.2. Application to the Published Dataset

The six selection methods were applied on a breast cancer dataset, with a fraction of a training data equal to 2/3. The number of selected probes was 54 for LASSO-cvl, 13 for LASSO-pcvl, 78 for Elastic Net, 42 for BSS Enet, 58 for CoxBoost, and 83 for RSF. The lists of probes selected by each method are given in Supp Table 1 and the intersections between them in Table 6.

**Figure 3:**
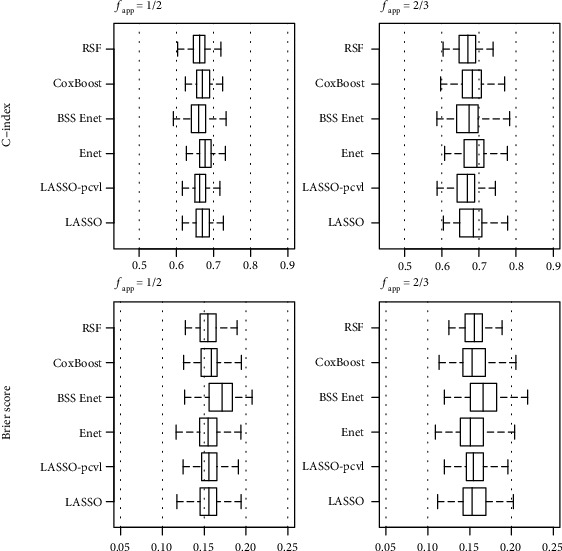
Prognostic performance for each selection method and fraction of the training data (*f*_app_) for the breast cancer dataset. (a) C-index and (b) Brier score associated to risk score.

RSF showed poor intersection with other methods. Among the probes selected by multiple methods, interesting targets in carcinogenesis could be identified that play a role in cell proliferation and apoptosis (CCDN1, MET, KIT, FGFR3,…), cell metabolism (BTG1, LPGAT1, CKB, ASNS,…), cell mobility and invasion (CDC42, PXDN, PFN2,…), and immunity (CD55, CXCL13, XBP1, HLA-DQB1,…). The same strategy of resampling was then applied to assess the impact of the training set on selection and performance. The number of selected genes increased when the training sample size increased. For a given sample size of the training set, more genes were selected by RSF than the other selection methods, and LASSO-pcvl was the most stringent method (Table 7). As per stability, 50% of the selected predictors had an occurrence frequency less than 5%. Discriminant capacities (e.g., C-index) were poor for all approaches, and BSS Enet tended to have the lowest performances (Figure 3).

## 4. Discussion

The main objective of this study was to provide an exhaustive comparison of popular variable selection methods and to offer recommendations for developing prognostic signatures. To our knowledge, this is the first comparison assessing the effect of the constitution of the training set on multiple criteria like stability, false discovery rates, and prognostic performances between classical extensions of the Cox proportional hazards model and random forest, the most commonly used nonparametric method in this context. Using simulated datasets and one real application, we investigated both variable selection and prognostic performances of each approach. Our main conclusion is that while there is great variability in the selection process, all variable selection methods provide good prognostic performance with a satisfactory C-index in most scenarios.

Using simulated datasets based on biologically plausible parameters, we evaluated the influence of both selection methods and sample sizes on selection and prognostic performances. Considering the different comparison criteria, LASSO-pcvl is the most advisable selection method since FDR is minimized whatever the sample size, with a moderate increase in FNR and prognostic performances similar to other selection methods. Conversely, RSF is the worst method in terms of performances with too many false positives selected in most scenarios and a lack of common genes with other approaches. This may be explained by the lack of a prefilter step in the “variable hunting” algorithm recommended for high-dimensional settings which does not limit the number of selected genes. A cross-validation to optimize this number, as for other selection methods, might improve RSF performances. On the other hand, RSF has been proposed to deal with complex relationships between covariates and survival and may perform worse with linear effects. Compared to classical Elastic Net, BSS Enet gives more satisfactory results in alternative scenarios with a lower FDR, despite a slightly higher FNR. The less variables are correlated with true predictors, the better BSS Enet performs, but this is an unlikely assumption in case of omics data. However, the systematic selection of false-positive genes under the null scenarios reduces the attractiveness of this approach. A sensitivity analysis examining the effects of a more stringent stability threshold on the risk score (70 and 80%) did not address this issue. Also, BSS does not improve stability in the selection process or prognostic performances compared to a single step in the present study. As expected, the discriminant capacity of the risk score obtained by the different methods increases when the number of true predictors associated with survival increases and when the number of false-positive genes selected is low. Due to overfitting, there is a decrease in discrimination performances on the validation dataset when the number of false positives increases. In the public dataset, we observed poor stability compared to simulation results and poor prognosis performance. Several reasons may explain these findings, for example, the small sample size. The instability may be explained by the fact that there is no unique solution due to the multidimensionality of the data [10]. Indeed, interactions and correlations between predictors in omics studies are often more complex than in our simulation study. Contrary to popular belief, it is important to note in our study that LASSO was able to simultaneously select correlated biomarkers simultaneously. Other simulations are necessary to determine the impact of the correlation structure on the selection of predictors in this approach.

This simulation study had various limitations. Firstly, other simulation strategies for time-to-event data could have been tested to check whether survival forests are not at too great a disadvantage in a Cox proportional hazards scenario. Secondly, in order to make a decision in clinical practice, it is often helpful to have threshold values that make it possible to determine different risk groups. Various techniques can be employed, like the minimum *p* value approach, cutting the continuous risk score into equal groups (according to the median or other quantiles), or strategies based on ROC curves. Further work is necessary to provide help in selecting the most adequate threshold depending on the clinical question. Then, the number of covariates in our study could be much larger that is often true for genomic data unless a prefilter step is applied; but from this initial work with “moderate” high-dimensional settings, it is clear that variable selection methods achieve even worse stability and performances when *p* > >>*n*. Finally, only one value was considered for the parameter of correlation in the autoregressive correlation structure, Nevertheless, we guess that a greater correlation may not be biologically relevant and that 0.6 is a reasonable choice for maximum correlation in our simulation study and a good compromise to evaluate statistical performance for biologically plausible scenarios.

In the context of precision medicine, gene signatures are usually developed independently from clinical factors, i.e., omics selection, development of a risk score and risk groups followed by adjustment on prognostic factors. This strategy does not make it possible to select genes with a prognostic value independent from a clinical value. Moreover, most of the gene signatures lose their significance after adjustment. To our knowledge, only one *in silico* study was interested in taking clinical factors into account during the gene selection step [33]. It could be of interest to evaluate the proposed approaches in terms of prognostic performance, but especially in terms of selected genes and associated signaling pathways. In breast cancer research, as over half of the genome is correlated with proliferation [8], it is quite easy to find several significant combinations of genes associated with clinical outcome. Nevertheless, identifying genes involved in signaling pathways other than cell cycles could highlight new therapeutic targets and improve prognostic models with both clinical and genomic data. Another important aspect is that selection methods investigated in this publication do not take into account knowledge of biomarker biological pathways [34]. The application on the breast dataset suggests that, despite some relevant selected genes previously described in the literature, many predictors are selected because of their correlation with true prognostic genes. Thus, an important issue in biomarker discovery is the true functional significance of the variables selected. To properly determine this, biologists, clinicians, and statisticians must work closely together to propose relevant gene signatures.

## Figures and Tables

**Table 1:** Characteristics of simulated datasets.

*N*	Events	Censoring rate (%)	*p*	*q*
500	448	10.4	1500	0
500	403	19.4	1500	12
500	362	27.6	1500	50
750	678	9.6	1500	0
750	617	17.7	1500	12
750	535	28.7	1500	50
1000	892	10.8	1500	0
1000	814	18.6	1500	12
1000	709	29.1	1500	50

**Table 2:** Number of selected predictors for each simulated dataset and selection method.

*N*	*q*	Training fraction	Number of selected predictors med (min–max)					
			LASSO-cvl	LASSO-pcvl	Elastic Net	BSS Enet	CoxBoost	RSF
500	0	1/2	0 (0-24)	1 (0-30)	0 (0-34)	9 (1-15)	0 (0-24)	71 (67-75)
		2/3	0 (0-21)	0 (0-31)	0 (0-24)	10 (4-20)	0 (0-24)	56 (52-59)
	12	1/2	25 (7-55)	11 (5-29)	33 (10-62)	22 (10-29)	26 (9-50)	71 (65-75)
		2/3	27 (10-55)	13 (7-26)	37 (13-75)	27 (19-35)	30 (10-50)	56 (52-60)
	50	1/2	62 (40-93)	44 (13-75)	83 (50-121)	44 (29-57)	57 (24-80)	75 (70-78)
		2/3	76 (57-105)	53 (34-72)	96 (71-127)	56 (47-66)	67 (41-103)	63 (57-68)

750	0	1/2	0 (0-18)	0 (0-33)	0 (0-23)	12 (6-22)	0 (0-29)	45 (42-49)
		2/3	0 (0-20)	0 (0-16)	0 (0-21)	16 (8-26)	0 (0-19)	22 (19-25)
	12	1/2	25 (12-52)	13 (7-25)	33 (14-57)	25 (14-36)	27 (12-53)	47 (44-51)
		2/3	24 (12-49)	11 (8-18)	28 (17-55)	28 (21-37)	26 (13-52)	25 (22-28)
	50	1/2	72 (51-93)	44 (27-68)	88 (65-118)	54 (46-69)	62 (36-85)	58 (53-61)
		2/3	80 (58-111)	46 (31-59)	96 (76-134)	64 (52-78)	69 (43-89)	37 (32-41)

1000	0	1/2	0 (0-45)	0 (0-36)	0 (0-45)	19 (10-34)	1 (0-40)	22 (20-25)
		2/3	1 (0-48)	1 (0-43)	1 (0-48)	27 (17-40)	2 (0-45)	6 (6-8)
	12	1/2	34 (11-79)	15 (8-28)	42 (18-89)	35 (24-52)	36 (17-62)	28 (25-32)
		2/3	42 (21-74)	15 (9-25)	50 (23-86)	48 (31-63)	44 (14-77)	9 (8-11)
	50	1/2	90 (70-124)	57 (34-78)	107 (87-139)	68 (58-89)	74 (54-114)	38 (34-43)
		2/3	101 (76-145)	57 (46-73)	120 (84-168)	80 (67-99)	79 (53-112)	18 (16-20)

**Table 3:** False discovery rates and false-negative rates for each simulated dataset and selection method.

*N*	*q*	Training fraction	FDR/FNR					
			LASSO-cvl	LASSO-pcvl	Elastic Net	BSS Enet	CoxBoost	RSF
500	0	1/2	0.43/.	0.51/.	0.45/.	1/.	0.49/.	1/.
		2/3	0.39/.	0.48/.	0.4/.	1/.	0.49/.	1/.
	12	1/2	0.62/0.29	0.32/0.36	0.69/0.19	0.56/0.24	0.65/0.3	0.92/0.56
		2/3	0.61/0.18	0.28/0.23	0.69/0.1	0.6/0.12	0.64/0.19	0.9/0.51
	50	1/2	0.51/0.39	0.35/0.45	0.56/0.3	0.3/0.39	0.47/0.41	0.83/0.74
		2/3	0.52/0.27	0.34/0.31	0.58/0.19	0.32/0.25	0.47/0.29	0.78/0.72

750	0	1/2	0.44/.	0.5/.	0.46/.	1/.	0.46/.	1/.
		2/3	0.23/.	0.42/.	0.24/.	1/.	0.38/.	1/.
	12	1/2	0.62/0.24	0.31/0.27	0.67/0.16	0.59/0.16	0.64/0.25	0.85/0.4
		2/3	0.57/0.21	0.19/0.23	0.62/0.12	0.63/0.11	0.61/0.2	0.65/0.29
	50	1/2	0.49/0.29	0.27/0.35	0.54/0.2	0.32/0.27	0.44/0.3	0.75/0.71
		2/3	0.48/0.17	0.2/0.26	0.54/0.1	0.33/0.15	0.4/0.2	0.54/0.66

1000	0	1/2	0.45/.	0.48/.	0.45/.	1/.	0.51/.	1/.
		2/3	0.55/.	0.57/.	0.56/.	1/.	0.62/.	1/.
	12	1/2	0.71/0.23	0.4/0.27	0.74/0.16	0.7/0.16	0.73/0.23	0.71/0.32
		2/3	0.75/0.19	0.36/0.23	0.78/0.12	0.77/0.1	0.77/0.19	0.22/0.4
	50	1/2	0.52/0.15	0.27/0.18	0.58/0.1	0.35/0.11	0.44/0.16	0.54/0.65
		2/3	0.56/0.1	0.24/0.13	0.61/0.06	0.42/0.07	0.44/0.12	0.15/0.7

**Table 4:** Gene frequency occurrence for each simulated dataset and selection method.

*N*	*q*	Training fraction	Occurrence frequency med (min–max) TP/FP					
			LASSO-cvl	LASSO-pcvl	Elastic Net	BSS Enet	CoxBoost	RSF
500	0	1/2	NA/1 (1-16)	NA/1 (1-19)	NA/1 (1-18)	NA/1 (1-26)	NA/1 (1-23)	NA/5 (1-71)
		2/3	NA/1 (1-18)	NA/1 (1-21)	NA/1 (1-22)	NA/2 (1-40)	NA/1 (1-23)	NA/4 (1-94)
	12	1/2	76 (3-100)/2 (1-30)	65 (2-100)/1 (1-13)	86 (19-100)/2 (1-37)	82 (19-100)/2 (1-28)	74 (1-100)/2 (1-31)	48 (9-94)/4 (1-89)
		2/3	92 (8-100)/2 (1-50)	86 (3-100)/2 (1-29)	97 (34-100)/2 (1-64)	97 (38-100)/2 (1-54)	92 (10-100)/2 (1-59)	52 (12-87)/4 (1-98)
	50	1/2	66 (1-99)/2 (1-53)	58 (1-100)/2 (1-37)	79 (1-100)/2 (1-67)	68 (3-100)/2 (1-34)	67 (1-99)/2 (1-46)	18 (1-87)/4 (1-62)
		2/3	89 (1-100)/2 (1-74)	82 (6-100)/2 (1-57)	94 (4-100)/3 (1-77)	91 (1-100)/2 (1-64)	87 (1-100)/2 (1-68)	22 (1-100)/3 (1-81)

750	0	1/2	NA/1 (1-14)	NA/1 (1-16)	NA/1 (1-17)	NA/1 (1-42)	NA/1 (1-10)	NA/3 (1-60)
		2/3	NA/1 (1-13)	NA/1 (1-18)	NA/1 (1-14)	NA/2 (1-71)	NA/1 (1-14)	NA/2 (1-86)
	12	1/2	94 (22-100)/2 (1-34)	90 (17-100)/1 (1-13)	99 (39-100)/2 (1-38)	98 (31-100)/1 (1-30)	94 (22-100)/2 (1-34)	59 (9-100)/3 (1-77)
		2/3	99 (17-100)/2 (1-44)	98 (12-100)/1 (1-15)	100 (35-100)/2 (1-48)	100 (28-100)/2 (1-42)	99 (14-100)/2 (1-47)	74 (11-100)/2 (1-60)
	50	1/2	78 (10-100)/2 (1-44)	72 (6-100)/1 (1-31)	90 (14-100)/2 (1-54)	84 (6-100)/2 (1-31)	76 (7-100)/2 (1-40)	21 (1-86)/3 (1-56)
		2/3	94 (13-100)/2 (1-68)	91 (4-100)/2 (1-48)	98 (28-100)/3 (1-78)	97 (12-100)/2 (1-57)	94 (11-100)/2 (1-56)	35 (1-100)/2 (1-55)

1000	0	1/2	NA/1 (1-20)	NA/1 (1-21)	NA/1 (1-22)	NA/2 (1-50)	NA/1 (1-26)	NA/2 (1-78)
		2/3	NA/1 (1-39)	NA/1 (1-43)	NA/1 (1-40)	NA/2 (1-80)	NA/1 (1-40)	NA/2 (1-80)
	12	1/2	97 (5-100)/2 (1-53)	97 (2-100)/1 (1-33)	100 (15-100)/2 (1-64)	99 (19-100)/2 (1-56)	98 (5-100)/2 (1-57)	70 (26-99)/2 (1-32)
		2/3	100 (6-100)/2 (1-84)	100 (2-100)/2 (1-49)	100 (16-100)/3 (1-87)	100 (21-100)/3 (1-89)	100 (5-100)/2 (1-88)	54 (28-100)/1 (1-14)
	50	1/2	98 (2-100)/2 (1-74)	93 (11-100)/1 (1-48)	99 (5-100)/3 (1-81)	99 (20-100)/2 (1-64)	95 (1-100)/2 (1-60)	30 (1-95)/2 (1-48)
		2/3	100 (2-100)/3 (1-93)	100 (6-100)/2 (1-77)	100 (5-100)/3 (1-97)	100 (2-100)/2 (1-92)	100 (7-100)/2 (1-86)	36 (1-86)/2 (1-22)

**Table 5:** Number of genes that overlaps between methods for the same samples and true-positive rates among common genes, for each simulated dataset and selection method.

*N*	*q*	Training fraction		Intersection (number of common genes/true-positive rates among common genes)				
				LASSO-pcvl	Elastic Net	BSS Enet	CoxBoost	RSF
500	12	1/2	LASSO-cvl	11 (5-29)/0.7 (0.35-1)	25 (7-53)/0.35 (0.15-1)	18 (7-27)/0.47 (0.26-1)	22 (7-40)/0.38 (0.17-1)	6 (1-12)/0.71 (0.2-1)
			LASSO-pcvl		11 (5-29)/0.7 (0.33-1)	11 (5-24)/0.71 (0.41-1)	11 (5-29)/0.7 (0.33-1)	4 (1-12)/0.86 (0.5-1)
			Elastic Net			20 (8-29)/0.45 (0.29-0.88)	24 (8-48)/0.35 (0.17-0.78)	7 (3-14)/0.67 (0.2-1)
			BSS Enet				18 (7-28)/0.44 (0.28-0.8)	7 (1-12)/0.75 (0.25-1)
			CoxBoost					6 (1-13)/0.71 (0.2-1)
	50	1/2	LASSO-cvl	42 (13-74)/0.64 (0.46-0.95)	62 (40-93)/0.49 (0.37-0.65)	40 (28-56)/0.71 (0.52-0.84)	53 (24-80)/0.55 (0.41-0.92)	12 (4-19)/0.79 (0.44-1)
			LASSO-pcvl		44 (13-74)/0.63 (0.45-0.95)	35 (13-48)/0.74 (0.59-0.95)	42 (13-72)/0.65 (0.45-0.95)	11 (3-18)/0.86 (0.56-1)
			Elastic Net			43 (29-57)/0.71 (0.52-0.82)	57 (24-79)/0.54 (0.38-0.92)	14 (5-21)/0.77 (0.37-1)
			BSS Enet				40 (22-53)/0.72 (0.53-0.95)	11 (2-18)/0.89 (0.57-1)
			CoxBoost					12 (4-20)/0.81 (0.5-1)

750	12	1/2	LASSO-cvl	13 (7-25)/0.69 (0.4-1)	25 (12-50)/0.36 (0.18-0.75)	19 (11-29)/0.47 (0.3-0.79)	23 (12-43)/0.39 (0.2-0.75)	7 (4-12)/0.83 (0.5-1)
			LASSO-pcvl		13 (7-25)/0.68 (0.4-1)	12 (6-23)/0.73 (0.43-1)	13 (7-24)/0.69 (0.38-1)	6 (3-10)/1 (0.6-1)
			Elastic Net			22 (11-33)/0.45 (0.3-0.75)	26 (12-50)/0.36 (0.2-0.75)	8 (4-14)/0.8 (0.54-1)
			BSS Enet				20 (11-33)/0.45 (0.3-0.82)	8 (4-12)/0.86 (0.43-1)
			CoxBoost					7 (3-12)/0.8 (0.57-1)
	50	1/2	LASSO-cvl	43 (27-68)/0.73 (0.5-0.91)	71 (51-90)/0.51 (0.37-0.69)	49 (39-65)/0.7 (0.55-0.85)	60 (36-79)/0.58 (0.43-0.81)	14 (6-22)/0.85 (0.67-1)
			LASSO-pcvl		44 (27-68)/0.73 (0.5-0.92)	40 (27-57)/0.79 (0.61-0.94)	42 (27-65)/0.74 (0.52-0.92)	12 (7-20)/0.93 (0.71-1)
			Elastic Net			52 (42-68)/0.68 (0.55-0.83)	62 (36-83)/0.56 (0.43-0.81)	16 (7-23)/0.83 (0.6-1)
			BSS Enet				47 (34-63)/0.71 (0.56-0.86)	14 (8-22)/0.9 (0.73-1)
			CoxBoost					14 (6-22)/0.88 (0.71-1)

1000	12	1/2	LASSO-cvl	15 (8-28)/0.59 (0.32-0.92)	34 (11-79)/0.28 (0.11-0.73)	26 (11-51)/0.35 (0.18-0.73)	31 (11-55)/0.29 (0.16-0.73)	8 (4-12)/0.89 (0.45-1)
			LASSO-pcvl		15 (8-28)/0.59 (0.32-0.92)	15 (8-27)/0.61 (0.33-0.92)	15 (8-28)/0.59 (0.32-0.92)	7 (4-11)/1 (0.62-1)
			Elastic Net			29 (14-52)/0.34 (0.17-0.61)	34 (16-62)/0.28 (0.14-0.53)	9 (4-12)/0.89 (0.42-1)
			BSS Enet				27 (13-44)/0.34 (0.2-0.59)	9 (6-12)/0.89 (0.5-1)
			CoxBoost					8 (4-11)/0.88 (0.6-1)
	50	1/2	LASSO-cvl	57 (34-77)/0.72 (0.52-0.97)	90 (70-122)/0.48 (0.35-0.61)	63 (53-88)/0.67 (0.52-0.78)	73 (54-101)/0.57 (0.45-0.78)	18 (13-24)/0.9 (0.71-1)
			LASSO-pcvl		57 (34-78)/0.72 (0.51-0.97)	53 (34-65)/0.78 (0.63-0.97)	56 (34-76)/0.73 (0.53-0.97)	17 (12-23)/0.94 (0.74-1)
			Elastic Net			66 (58-89)/0.66 (0.53-0.76)	74 (54-106)/0.56 (0.43-0.78)	20 (14-25)/0.88 (0.71-1)
			BSS Enet				60 (50-84)/0.7 (0.56-0.81)	19 (13-24)/0.94 (0.79-1)
			CoxBoost					18 (12-24)/0.94 (0.71-1)

500	12	2/3	LASSO-cvl	13 (7-25)/0.71 (0.4-1)	26 (10-55)/0.38 (0.15-0.8)	20 (9-31)/0.45 (0.3-0.8)	24 (10-46)/0.4 (0.17-0.8)	7 (3-11)/0.75 (0.43-1)
			LASSO-pcvl		13 (7-26)/0.71 (0.38-1)	13 (7-21)/0.72 (0.48-1)	13 (7-25)/0.71 (0.4-1)	5 (2-10)/1 (0.6-1)
			Elastic Net			23 (11-33)/0.43 (0.31-0.82)	28 (10-48)/0.34 (0.17-0.71)	7 (4-13)/0.71 (0.4-1)
			BSS Enet				21 (9-32)/0.44 (0.31-0.78)	7 (4-11)/0.8 (0.5-1)
			CoxBoost					7 (3-10)/0.71 (0.43-1)
	50	2/3	LASSO-cvl	52 (33-71)/0.65 (0.51-0.85)	76 (57-104)/0.49 (0.34-0.61)	51 (43-62)/0.68 (0.56-0.81)	65 (41-93)/0.55 (0.35-0.68)	14 (7-21)/0.83 (0.58-1)
			LASSO-pcvl		53 (34-72)/0.65 (0.51-0.85)	46 (34-57)/0.73 (0.61-0.92)	51 (33-65)/0.66 (0.56-0.87)	13 (7-19)/0.9 (0.64-1)
			Elastic Net			54 (45-66)/0.69 (0.56-0.82)	66 (41-99)/0.54 (0.34-0.69)	16 (10-23)/0.8 (0.48-1)
			BSS Enet				50 (37-60)/0.7 (0.6-0.82)	14 (7-20)/0.92 (0.61-1)
			CoxBoost					14 (7-21)/0.87 (0.62-1)

750	12	2/3	LASSO-cvl	11 (8-18)/0.82 (0.5-1)	23 (12-49)/0.42 (0.2-0.79)	20 (11-33)/0.5 (0.3-0.92)	21 (12-47)/0.45 (0.21-0.77)	8 (5-11)/1 (0.73-1)
			LASSO-pcvl		11 (8-18)/0.82 (0.5-1)	11 (8-17)/0.83 (0.53-1)	11 (8-18)/0.82 (0.5-1)	7 (5-10)/1 (0.86-1)
			Elastic Net			22 (13-35)/0.46 (0.29-0.85)	24 (13-49)/0.41 (0.2-0.71)	8 (5-13)/1 (0.69-1)
			BSS Enet				20 (12-32)/0.47 (0.28-0.83)	8 (5-12)/1 (0.73-1)
			CoxBoost					8 (5-12)/1 (0.7-1)
	50	2/3	LASSO-cvl	46 (31-59)/0.8 (0.62-0.97)	80 (58-111)/0.52 (0.39-0.72)	58 (46-70)/0.69 (0.59-0.85)	68 (43-87)/0.59 (0.45-0.84)	16 (12-21)/0.94 (0.76-1)
			LASSO-pcvl		46 (31-59)/0.8 (0.62-0.97)	44 (30-54)/0.83 (0.69-0.97)	46 (31-58)/0.8 (0.65-0.97)	14 (9-20)/1 (0.84-1)
			Elastic Net			62 (51-78)/0.68 (0.59-0.84)	69 (43-89)/0.59 (0.44-0.84)	17 (14-23)/0.94 (0.74-1)
			BSS Enet				55 (40-67)/0.71 (0.6-0.87)	16 (12-22)/0.94 (0.81-1)
			CoxBoost					15 (12-21)/0.94 (0.8-1)

1000	12	2/3	LASSO-cvl	15 (9-25)/0.62 (0.4-1)	42 (20-74)/0.24 (0.12-0.5)	33 (19-55)/0.29 (0.16-0.53)	37 (14-67)/0.25 (0.15-0.57)	7 (3-9)/1 (0.83-1)
			LASSO-pcvl		15 (9-25)/0.62 (0.4-1)	14 (9-25)/0.62 (0.4-1)	14 (9-25)/0.62 (0.4-1)	6 (3-9)/1 (0.8-1)
			Elastic Net			38 (21-59)/0.28 (0.18-0.52)	41 (14-70)/0.23 (0.14-0.57)	7 (4-10)/1 (0.83-1)
			BSS Enet				35 (14-53)/0.28 (0.17-0.57)	7 (4-10)/1 (0.75-1)
			CoxBoost					7 (4-9)/1 (0.83-1)
	50	2/3	LASSO-cvl	57 (45-73)/0.76 (0.6-0.91)	100 (75-144)/0.45 (0.31-0.59)	73 (59-89)/0.61 (0.5-0.74)	78 (53-110)/0.56 (0.39-0.77)	15 (11-18)/1 (0.88-1)
			LASSO-pcvl		57 (46-73)/0.75 (0.6-0.91)	55 (45-68)/0.79 (0.63-0.91)	55 (45-71)/0.76 (0.61-0.91)	15 (11-18)/1 (0.92-1)
			Elastic Net			78 (65-93)/0.59 (0.5-0.72)	79 (53-112)/0.55 (0.38-0.77)	16 (12-18)/1 (0.81-1)
			BSS Enet				67 (53-82)/0.66 (0.53-0.83)	15 (11-18)/1 (0.92-1)
			CoxBoost					15 (11-18)/1 (0.92-1)

**Table 6:** Number of genes that overlap between methods for breast cancer dataset (*sample* results).

	LASSO-cvl	LASSO-pcvl	Elastic Net	BSS Enet	CoxBoost	RSF
LASSO-cvl	*54*	10	54	32	49	7
LASSO-pcvl		*13*	12	9	12	2
Elastic Net			*78*	38	56	9
BSS Enet				*42*	34	7
CoxBoost					*58*	8
RSF						*83*

**Table 7:** Number of selected predictors and gene frequency occurrence for breast cancer dataset and each selection method (resampling results).

Training fraction	Number of selected predictors med (min–max)					
	LASSO-cvl	LASSO-pcvl	Elastic Net	BSS Enet	CoxBoost	RSF
1/2	26 (4-71)	11 (0-59)	47 (11-100)	30 (17-49)	26 (0-56)	82 (71-93)
2/3	40 (6-70)	14 (4-53)	58 (14-104)	41 (31-62)	40 (5-70)	85 (72-93)
	Occurrence frequency med (min–max)					
1/2	2 (1-65)	2 (1-57)	2 (1-73)	2 (1-77)	2 (1-64)	5 (1-57)
2/3	3 (1-92)	2 (1-73)	3 (1-95)	3 (1-98)	3 (1-91)	5 (1-61)

## Data Availability

The breast cancer dataset was extracted from GitHub (http://github.com/Oncostat/biospear/). Simulated datasets are available from the corresponding authors on reasonable request.

## Supplementary Materials

Supplementary Materials Supp Table 1: list of selected probes and associated genes in breast cancer dataset (sample results).
