# Supplementary material for: Comparison of Variable Selection Methods for Time-to-Event Data in High-Dimensional Settings
Source: Comput Math Methods Med. 2020 Jul 1;2020:6795392. doi: 10.1155/2020/6795392 (PMC7350178; doi:10.1155/2020/6795392)
Supplement: Supplementary Materials — Supp Table 1: list of selected probes and associated genes in breast cancer dataset (sample results). [file 6795392.f1.pdf]

Supplementary Table 1. List of selected probes and associated genes in breast cancer dataset (sample results)

LASSO-cvl		LASSO-pcvl		Elastic net		BSS enet		Coxboost		RSF	
200800_s_at	HSPA1A///HSPA1B	200670_at	XBP1	200670_at	XBP1	200800_s_at	HSPA1A///HSPA1B	200670_at	XBP1	200632_s_at	NDRG1
200920_s_at	BTG1	201195_s_at	SLC7A5	200800_s_at	HSPA1A///HSPA1B	201195_s_at	SLC7A5	200800_s_at	HSPA1A///HSPA1B	200800_s_at	HSPA1A///HSPA1B
200985_s_at	CD59	201925_s_at	CD55	200884_at	CKB	201325_s_at	EMP1	200920_s_at	BTG1	200989_at	HIF1A
201195_s_at	SLC7A5	202769_at	CCNG2	200920_s_at	BTG1	201912_s_at	GSPT1	200985_s_at	CD59	201131_s_at	CDH1
201325_s_at	EMP1	203510_at	MET	200985_s_at	CD59	201925_s_at	CD55	201195_s_at	SLC7A5	201160_s_at	YBX3
201876_at	PON2	203929_s_at	MAPT	201195_s_at	SLC7A5	202203_s_at	AMFR	201287_s_at	SDC1	201195_s_at	SLC7A5
201911_s_at	FARP1	205862_at	GREB1	201287_s_at	SDC1	202651_at	LPGAT1	201876_at	PON2	201286_at	SDC1
201912_s_at	GSPT1	206042_x_at	SNRPN///SNURF	201325_s_at	EMP1	202824_s_at	TCEB1	201912_s_at	GSPT1	201324_at	EMP1
201925_s_at	CD55	212492_s_at	KDM4B	201876_at	PON2	202888_s_at	ANPEP	201925_s_at	CD55	201325_s_at	EMP1
202035_s_at	SFRP1	212593_s_at	PDCD4	201911_s_at	FARP1	203243_s_at	PDLIM5	202035_s_at	SFRP1	201397_at	PHGDH
202203_s_at	AMFR	217838_s_at	EVL	201912_s_at	GSPT1	203510_at	MET	202203_s_at	AMFR	201589_at	SMC1A
202314_at	CYP51A1///LRRD1	219922_s_at	LTBP3	201925_s_at	CD55	203814_s_at	NQO2	202314_at	CYP51A1///LRRD1	201849_at	BNIP3
202769_at	CCNG2	221589_s_at	ALDH6A1	202035_s_at	SFRP1	203928_x_at	MAPT	202651_at	LPGAT1	201876_at	PON2
202824_s_at	TCEB1			202203_s_at	AMFR	203953_s_at	CLDN3	202769_at	CCNG2	202016_at	MEST
203153_at	IFIT1			202314_at	CYP51A1///LRRD1	204379_s_at	FGFR3	202824_s_at	TCEB1	202454_s_at	ERBB3
203243_s_at	PDLIM5			202351_at	ITGAV	204641_at	NEK2	203153_at	IFIT1	202600_s_at	NRIP1
203510_at	MET			202651_at	LPGAT1	204942_s_at	ALDH3B2	203243_s_at	PDLIM5	202856_s_at	SLC16A3
203814_s_at	NQO2			202769_at	CCNG2	204992_s_at	PFN2	203510_at	MET	202862_at	FAH
203910_at	ARHGAP29			202824_s_at	TCEB1	205030_at	FABP7	203814_s_at	NQO2	202870_s_at	CDC20
204379_s_at	FGFR3			203153_at	IFIT1	205051_s_at	KIT	203910_at	ARHGAP29	202887_s_at	DDIT4
204992_s_at	PFN2			203243_s_at	PDLIM5	205242_at	CXCL13	204379_s_at	FGFR3	202888_s_at	ANPEP
205030_at	FABP7			203510_at	MET	205573_s_at	SNX7	204992_s_at	PFN2	202890_at	MAP7
205047_s_at	ASNS			203814_s_at	NQO2	205862_at	GREB1	205030_at	FABP7	203126_at	IMPA2
205242_at	CXCL13			203910_at	ARHGAP29	206042_x_at	SNRPN///SNURF	205047_s_at	ASNS	203335_at	PHYH
205862_at	GREB1			203929_s_at	MAPT	206373_at	ZIC1	205051_s_at	KIT	203510_at	MET
206042_x_at	SNRPN///SNURF			204070_at	RARRES3	207414_s_at	PCSK6	205242_at	CXCL13	203535_at	S100A9
206560_s_at	MIA			204379_s_at	FGFR3	207469_s_at	PIR	205862_at	GREB1	203638_s_at	FGFR2
207414_s_at	PCSK6			204641_at	NEK2	208083_s_at	ITGB6///LOC100505984	206042_x_at	SNRPN///SNURF	203710_at	ITPR1
207469_s_at	PIR			204992_s_at	PFN2	208451_s_at	C4A///C4B///C4B_2	206373_at	ZIC1	203764_at	DLGAP5
208083_s_at	ITGB6///LOC100505984			205030_at	FABP7	208727_s_at	CDC42	206560_s_at	MIA	203954_x_at	CLDN3
208451_s_at	C4A///C4B///C4B_2			205047_s_at	ASNS	209301_at	CA2	207414_s_at	PCSK6	203962_s_at	NEBL

208490_x_at	HIST1H2BF	205051_s_at	KIT	209357_at	CITED2	207469_s_at	PIR	203973_s_at	CEBPD
208727_s_at	CDC42	205120_s_at	SGCB	212492_s_at	KDM4B	208451_s_at	C4A///C4B///C4B_2	204014_at	DUSP4
209301_at	CA2	205242_at	CXCL13	212593_s_at	PDCD4	208490_x_at	HIST1H2BF	204041_at	MAOB
209357_at	CITED2	205573_s_at	SNX7	213050_at	COBL	208727_s_at	CDC42	204162_at	NDC80
209369_at	ANXA3	205862_at	GREB1	214079_at	DHRS2	209301_at	CA2	204351_at	S100P
210387_at	HIST1H2BG	205890_s_at	GABBR1///UBD	218331_s_at	FAM208B	209357_at	CITED2	204400_at	EFS
210397_at	DEFB1	206042_x_at	SNRPN///SNURF	218694_at	ARMCX1	209369_at	ANXA3	204862_s_at	NME3
210512_s_at	VEGFA	206373_at	ZIC1	219850_s_at	EHF	210387_at	HIST1H2BG	204863_s_at	IL6ST
212013_at	PXDN	206560_s_at	MIA	219922_s_at	LTBP3	210397_at	DEFB1	204913_s_at	SOX11
212492_s_at	KDM4B	207414_s_at	PCSK6	221589_s_at	ALDH6A1	211654_x_at	HLA-DQB1	204992_s_at	PFN2
212563_at	BOP1	207469_s_at	PIR	221675_s_at	CHPT1	212013_at	PXDN	205916_at	S100A7
212593_s_at	PDCD4	207980_s_at	CITED2			212492_s_at	KDM4B	206373_at	ZIC1
212730_at	SYNM	208083_s_at	ITGB6///LOC100505984			212496_s_at	KDM4B	206834_at	HBD///HBD
212998_x_at	HLA-DQB1	208451_s_at	C4A///C4B///C4B_2			212563_at	BOP1	207076_s_at	ASS1
213050_at	COBL	208490_x_at	HIST1H2BF			212593_s_at	PDCD4	207843_x_at	CYB5A
214079_at	DHRS2	208711_s_at	CCND1			212730_at	SYNM	208051_s_at	PAIP1
218694_at	ARMCX1	208727_s_at	CDC42			212998_x_at	HLA-DQB1	208654_s_at	CD164
219850_s_at	EHF	209301_at	CA2			213050_at	COBL	208933_s_at	LGALS8
219922_s_at	LTBP3	209357_at	CITED2			214079_at	DHRS2	208941_s_at	
221589_s_at	ALDH6A1	209369_at	ANXA3			217838_s_at	EVL	209008_x_at	KRT8
221675_s_at	CHPT1	209466_x_at	PTN			218331_s_at	FAM208B	209194_at	CETN2
221795_at	NTRK2	209835_x_at	CD44			218694_at	ARMCX1	209603_at	GATA3
221796_at	NTRK2	210239_at	IRX5			219850_s_at	EHF	209610_s_at	SLC1A4
		210387_at	HIST1H2BG			219922_s_at	LTBP3	209699_x_at	AKR1C2
		210397_at	DEFB1			221589_s_at	ALDH6A1	210239_at	IRX5
		210512_s_at	VEGFA			221675_s_at	CHPT1	210397_at	DEFB1
		211654_x_at	HLA-DQB1			221796_at	NTRK2	211074_at	---
		212013_at	PXDN					212063_at	CD44
		212492_s_at	KDM4B					212239_at	PIK3R1
		212510_at	GPD1L					212442_s_at	CERS6
		212563_at	BOP1					212496_s_at	KDM4B
		212593_s_at	PDCD4					212531_at	LCN2
		212730_at	SYNM					212771_at	

212998_x_at	HLA-DQB1	213357_at	GTF2H5
213050_at	COBL	213587_s_at	ATP6V0E2
214079_at	DHRS2	214370_at	S100A8
215440_s_at	BEX4	214709_s_at	KTN1
218162_at	OLFML3	217234_s_at	EZR
218331_s_at	FAM208B	218027_at	MRPL15
218694_at	ARMCX1	218194_at	REXO2
218847_at	IGF2BP2	218640_s_at	PLEKHF2
219850_s_at	EHF	218773_s_at	MSRB2
219922_s_at	LTBP3	219148_at	
221589_s_at	ALDH6A1	219192_at	UBAP2
221675_s_at	CHPT1	219359_at	ATHL1
221795_at	NTRK2	219681_s_at	RAB11FIP1
221796_at	NTRK2	219983_at	HRASLS
		220414_at	CALML5
		220625_s_at	ELF5
		33323_r_at	SFN
		74694_s_at	RABEP2
		AFFX.r2.Ec.bioC.5_at	---
